# Long-term Follow-up of Patients With Hernia Using the Hernia-Specific Quality-of-Life Mobile App: Feasibility Questionnaire Study

**DOI:** 10.2196/39759

**Published:** 2022-10-19

**Authors:** Ching-Shui Huang, Feng-Chuan Tai, Heng-Hui Lien, Jia-Uei Wong, Chi-Cheng Huang

**Affiliations:** 1 Division of General Surgery Department of Surgery Cathay General Hospital Taipei Taiwan; 2 School of Medicine College of Medicine Taipei Medical University Taipei Taiwan; 3 School of Medicine College of Medicine Fu-Jen Catholic University New Taipei Taiwan; 4 Department of Surgery Taipei Veterans General Hospital Taipei Taiwan; 5 Comprehensive Breast Health Center Taipei Veterans General Hospital Taipei Taiwan; 6 Institute of Epidemiology and Preventive Medicine National Taiwan University Taipei Taiwan

**Keywords:** hernia, mobile app, quality of life, Hernia-Specific Quality-of-Life (HERQL), mobile health, mHealth, app, self-management

## Abstract

**Background:**

Hernia repair is one of the most common surgical procedures; however, the long-term outcomes are seldom reported due to incomplete follow-up.

**Objective:**

The aim of this study was to examine the use of a mobile app for the long-term follow-up of hernia recurrence, complication, and quality-of-life perception.

**Methods:**

A cloud-based corroborative system drove a mobile app with the HERQL (Hernia-Specific Quality-of-Life) questionnaire built in. Patients who underwent hernia repair were identified from medical records, and an invitation to participate in this study was sent through the post.

**Results:**

The response rate was 11.89% (311/2615) during the 1-year study period, whereas the recurrence rate was 1.0% (3/311). Causal relationships between symptomatic and functional domains of the HERQL questionnaire were indicated by satisfactory model fit indices and significant regression coefficients derived from structural equational modeling. Regarding patients’ last hernia surgeries, 88.7% (276/311) of the patients reported them to be satisfactory or very satisfactory, 68.5% (213/311) of patients reported no discomfort, and 61.1% (190/311) of patients never experienced mesh foreign body sensation. Subgroup analysis for the most commonly used mesh repairs found that mesh plug repair inevitably resulted in worse symptoms and quality-of-life perception from the group with groin hernias.

**Conclusions:**

The mobile app has the potential to enhance the quality of care for patients with hernia and facilitate outcomes research with more complete follow-up.

## Introduction

Hernia repair is one of the most common surgical procedures performed each day worldwide, which can be traced back to the era of ancient Egypt [1]. With the development of prosthetic mesh and tension-free techniques, the recurrence rate following hernia repair has been reduced drastically [2-8]. For abdominal wall (ie, ventral or incisional) hernias, the recurrence rate can be reduced from 50% of primary repair to 10% to 23% with a prosthetic mesh [9]. On the other hand, the open anterior approach with mesh repair has replaced the Shouldice procedure as the standard operation with the benefits of shorter hospital stays, lower recurrences, and decreased postoperative pain; the recurrence rate was even lower (<1%) for groin hernia repair [10].

With reduced herniorrhaphy failures, outcomes research of hernia surgery should concentrate on postoperative quality of life and complications such as chronic pain [11,12]. The long-term outcomes of herniorrhaphy, however, have not been thoroughly evaluated. Loss of follow-up and poor compliance from patients result in a biased evaluation of recurrence, complication, and patients’ subjective quality of life [13,14].

To understand treatment outcomes, several quality-of-life instruments specific to hernia disease, such as the Carolinas Comfort Scale (CCS), the Hernia-Related Quality-of-Life Survey, the European Abdominal Wall Hernia Quality-of-Life Scores, the Core Outcome Measures Index adapted for patients with hernia, the Inguinal Pain Questionnaire, and the Brief Pain Inventory, have been developed and reported [15-20]. In the past few years, we have developed and validated an instrument—the Hernia-Specific Quality-of-Life (HERQL) questionnaire—for both groin and abdominal wall hernias. The questionnaire comprises a 4-item summative pain score measuring pain and discomfort resulting from various strenuous activities. Both symptomatic and functional domains, as well as postoperative satisfaction, are assessed with additional evaluations of hernia-related complications [21,22]. The validation study was conducted among 183 Taiwanese patients with groin hernia and 386 assessments; the internal reliability of the multi-item summative pain score was satisfactory (Cronbach α=.85). Criterion validity was evidenced by substantial to moderate correlations of the HERQL questionnaire with the five-level EQ-5D in pain/discomfort and health impact subscales [23]. Clinical validity was ascertained from worse hernia protrusion, pain during mild to heavy exercise, activity restriction, and health impairment scores reported from preoperative compared to postoperative patients. Clinical responsiveness was indicated by the time effect of –1.63 in the summative pain score from repeated measures [21].

The HERQL questionnaire targets both abdominal wall and inguinal hernias, traditional open and minimally invasive surgeries, and various mesh materials [21,22]. One merit of using the HERQL questionnaire for hernia outcomes research is the determination of the causal relationship between formative symptomatic scales and reflective functional indicators, which is elaborated through the pathway analysis of structural equation modeling (SEM) [24-26].

As previously mentioned, low compliance and high loss-of-follow-up rates among patients with hernia heavily compromised outcome evaluation of herniorrhaphy, especially when long-term outcomes were pursued. Indeed, there remains an unmet need to understand the true recurrence rate of hernia surgery, as well as the associated complications and subjective well-being [27]. To overcome these limitations, we purposed a novel mobile app to enhance the follow-up and outcomes research of patients with hernia. Indeed, mobile devices have been advocated as an effective tool for administration of screening tests among populations, from school-age children to adults; there is comprehensive evidence regarding the effectiveness of smartphone-based mobile apps for follow-up among surgical patients, especially for those who have undergone hernia repair [28-30].

## Methods

### Study Design and Subjects

We invited patients who completed hernia repair at our hospital to participate in this study. Index cases were identified from medical records. Both groin and abdominal hernias were eligible, with the latter comprising primary ventral and incisional hernias. The latest hernia surgery should have been performed at least 1 year prior to the starting date of the study. The enrollment period was between April 1, 2016, and March 31, 2017. Identified cases were contacted through the post using addresses from the medical charts.

A preset combination of a unique ID and password was sent to each invitee concurrently, and electronically signed informed consent was obtained with the mobile app through the built-in signature module (see Mobile App section). A copy of the informed consent document was sent to the email address provided by each invitee for reference.

### The HERQL Questionnaire

The HERQL questionnaire has been described elsewhere [21]. In brief, the HERQL questionnaire comprises a 4-item summative pain score measuring pain and discomfort resulting from various strenuous activities (ie, rest or mild, moderate, or heavy activities). In the meantime, symptomatic burden and functional domains, as well as postoperative satisfaction and potential complications, were assessed concurrently.

Pain and activity restriction due to pain or discomfort were rated on an 11-point Likert-type scale, ranging from 0 to 10, for each item, while symptomatic and functional domains (ie, hernia protrusion, analgesic usage, hernia’s impact on health, economic burden, and subjective quality of life/global health) were evaluated using a 5-point Likert-type scale. An auxiliary postoperative module, also equipped with 5-point Likert-type scales, was designed for potential complications following hernia repairs; these items included mesh foreign body sensation, severity of complications, overall satisfaction with hernia repair, confidence that hernia will not recur, and quality-of-life improvement by hernia repair. All scales were arranged with higher values representing compromised functionality or worse symptoms. The causal and indicator variables model proposed by Fayers et al [24,25] and Boehmer et al [26] formed the basis of HERQL questionnaire structure [21,22]. Elaboration on causal-indicator duality recognized one-way causal effects of symptomatic scales on functional domains, but not vice versa [16]. The content of the HERQL instrument is displayed in Textbox 1.

**Content of the Hernia-Specific Quality-of-Life instrument.**

**Summative pain score measures pain and discomfort resulting from rest or mild, moderate, or heavy activities:**

- 11-point Likert-type scale
- Q01, Q03, Q04, and Q05 (Q: question)

**Activity restriction due to pain or discomfort:**

- 11-point Likert-type scale
- Q09

**Symptomatic domains:**

- 5-point Likert-type scale
- Hernia protrusion: Q02
- Analgesic use: Q08

**Functional domains:**

- 5-point Likert-type scale
- Hernia’s impact on health: Q11
- Economic burden: Q12
- Subjective quality-of-life/global health perception: Q13

**Postoperative module:**

- 5-point Likert-type scale
- Mesh foreign body sensation: Q15
- Severity of complications: Q17
- Overall satisfaction with hernia repair: Q18
- Confidence that hernia will never recur: Q19
- Quality-of-life improvement by hernia repair: Q20

### Mobile App

**Figure 1:**
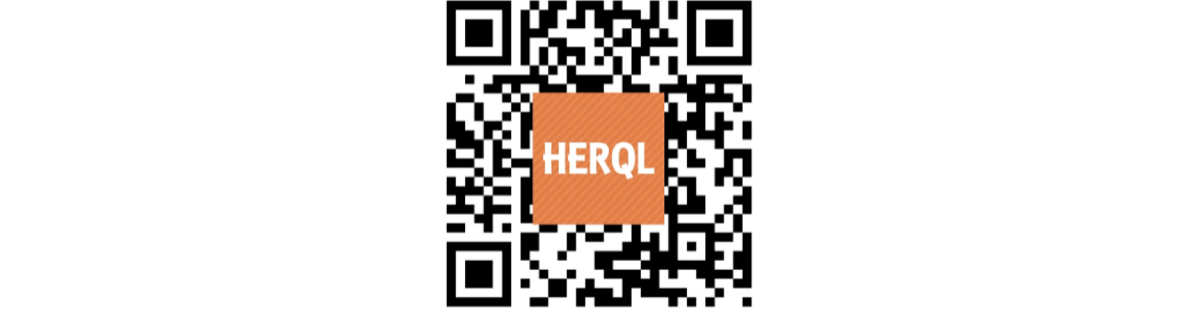
QR code for the HERQL questionnaire mobile app. HERQL: Hernia-Specific Quality-of-Life.

The mobile app version of the HERQL questionnaire assessing patients’ quality of life was ready for log-on for index cases identified from medical records, and an invitation was sent by post to those who had undergone hernia repairs at our institute at least 1 year before the study began. Both Android and iOS platforms were supported; a URL that linked to an online Google Docs–based questionnaire was provided as an alternative for those not equipped with a smartphone but who had internet access [31]. Figure 1 shows the QR code for the HERQL questionnaire mobile app, and Figure S1 in Multimedia Appendix 1 shows screenshots from an iOS-based device capturing all steps from log-on to the end of the survey. A corresponding Google website was established for communication and educational purposes (Figure S2 in Multimedia Appendix 1 [32]). The mobile app system was developed in cooperation with SynerFUN Technology Corporation, based in Hsinchu City, Taiwan.

The mobile app was driven by a cloud-based corroborative system. The system comprised a data management and storage subunit, as well as a security information subunit. Patients with hernia could administer HERQL, to report their quality-of-life and outcomes following hernia repair. The platform provided an easy and efficient way for patients to report any discomfort to their surgeons, which was designed to enhance the long-term follow-up and compliance of patients with hernia. A built-in signature module was developed to facilitate acquisition of electronically signed informed consent (Figure S3 in Multimedia Appendix 1).

### Statistical Methods

Subgroup comparisons were conducted between the most commonly used mesh materials from the group with groin hernias. Quality-of-life scores were treated as continuous variables; the Student *t* test was used for between-group comparisons. P values less than .05 were considered statistically significant.

The SEM concept was evaluated with the following model fit indices. Goodness of fit was evaluated by the ratio of chi-square to the degrees of freedom, and a ratio of less than 3 indicated a good fit of the hypothesized construct to the experimental data. Additional fit indices included the goodness-of-fit index (GFI; >0.90), the adjusted GFI (>0.80), the standardized root mean square residual (<0.1), the comparative fit index (>0.9), and the root mean square error of approximation (RMSEA; <0.08). All variances for latent factors were determined to be the ones used for model identification purposes.

### Ethics Approval

Human subject research ethics review and approval was in accordance with the regulation of the Institutional Review Board of Cathay General Hospital (protocol number: CGH-P102069). Written informed consent using electronic signatures was obtained from all participants, and all study subjects were compensated by post with a remuneration of 200 New Taiwan Dollars (approximately US $7 in 2016). Analyses were conducted after all data were anonymized for privacy and confidentiality protection.

## Results

### Study Population

During the 1-year study period, 2615 patients who had their hernia repaired at our institute were invited to participate in the study via the post. Among them, 2245 (85.85%) were male and 370 (14.15%) were female. The mean age was 60 (SD 15) years (median 62; range 18-95 years). The response rate was 11.89%: 311 patients followed the instructions, with successful log-on, and completed the HERQL survey. There were 93 (29.9%) abdominal wall (ie, incisional and ventral) hernias, 202 (65.0%) groin hernias, and 16 (5.14%) patients had both. The earliest hernia repair took place more than 13 years ago (mean 5.5, SD 2.7 years; median 5.4, range 1-13.6 years). Most responders were within 5 years of hernia repairs. Table 1 shows the types of prosthetic mesh adopted during herniorrhaphy.

**Table 1:** Types of prosthetic mesh used for hernia repairs.

Mesh type	Groin hernia (n=218^a^), n (%)	Abdominal wall hernia (n=109^a^), n (%)
Composix or Ventrio	0 (0)	38 (34.9)
Kugel or modified Kugel	60 (27.5)	11 (10.1)
Prolene Hernia System or Ultrapro Hernia System	57 (26.1)	11 (10.1)
Parietex	2 (0.9)	0 (0)
Mesh plug	77 (35.3)	9 (8.3)
Laparoscopy	13 (6.0)	2 (1.8)
Others	9 (4.1)	38 (34.9)

^a^This number includes 16 patients with both groin and abdominal wall hernias.

### SEM Concept of the HERQL Questionnaire

**Figure 2:**
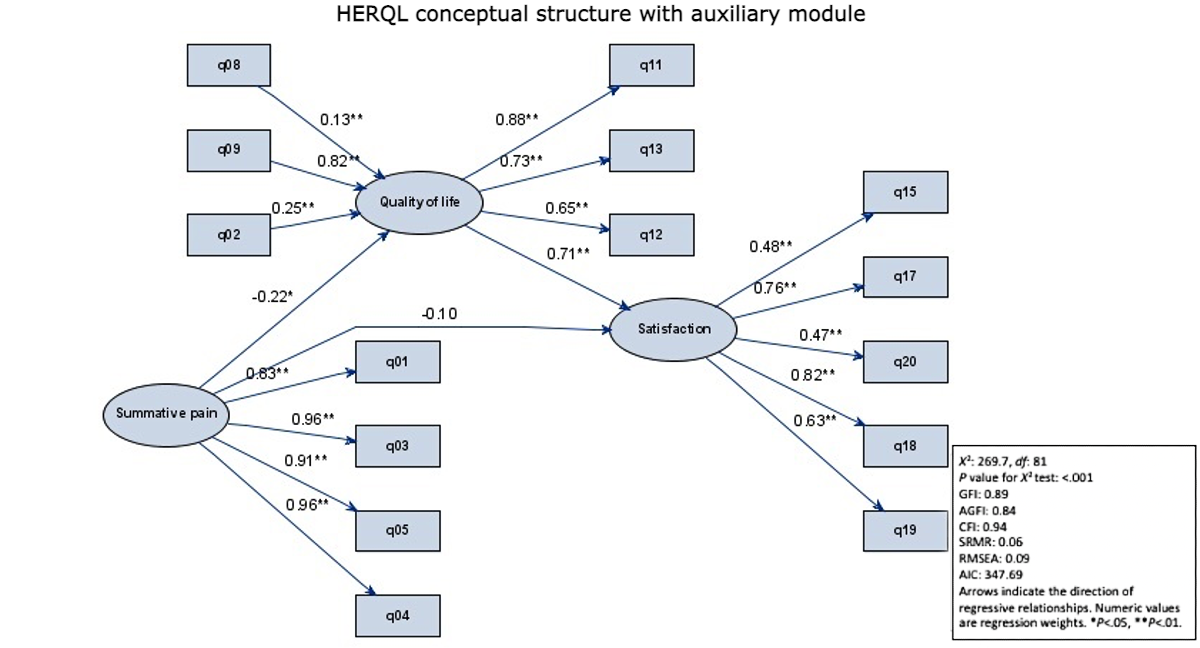
Conceptual structure of HERQL (Hernia-Specific Quality-of-Life) with the auxiliary post-operative module. Circles: latent factors, rectangles: measured variables (questionnaire items). AGFI: adjusted goodness-of-fix index; AIC: Akaike's information criterion; CFI: comparative fit index ; GFI: goodness-of-fix index; Q01: pain at rest, Q02: hernia protrusion, Q03: pain from mild activity, Q04: pain from moderate activity, Q05: pain from heavy activity, Q08: analgesic usage, Q09: activity restriction, Q11: hernia’s impact on health, Q12: economic burden, Q13: quality-of-life/global health, Q15: foreign body sensation, Q17: complication severity, Q18: overall satisfaction, Q19: confidence in hernia repair, Q20: quality-of-life improvement by hernia repair; RMSEA: root mean square error of approximation; SRMR: standardized root mean square residual. Arrows indicate the direction of regressive relationships. Numeric values are regression weights. *P<.05, **P<.01.

Figure 2 shows the conceptual structure of the HERQL questionnaire with the postoperative module. Goodness of fit (*χ*^2^/*df*=3.3) was slightly deviated from a good fit of the hypothesized construct to the experimental data. In large data sets with hundreds of samples, this deviation is not uncommon and was acceptable. Other indices supported the concept of the HERQL questionnaire for long-term follow-up of patients with hernia, with slight deviations of GFI (0.89) and RMSEA (0.09). Most importantly, the causal relationship between the summative pain score and the quality-of-life latent factor was indicated by a significant –0.22 regression coefficient, whereas the significant and positive 0.71 regression coefficient was reported for the causal relationship between the quality-of-life and postoperative satisfactory latent factors.

### Long-term Follow-Up of Hernia Repairs

The overall recurrence rate among 311 participants was 1.0% (n=3; Q19), and 88.7% (n=276) of participants rated their last hernia repair as satisfactory or very satisfactory (Q18). Approximately 70% of invitees (n=213, 68.5%) reported no discomfort with their hernia repair (Q16), and 61.1% (n=190) never experienced mesh foreign body sensation (Q15). Quality-of-life improvement following hernia repairs was ascertained in 90.4% (n=281) of enrolled subjects (Q20).

### Subgroup Analysis

Comparisons were conducted between 77 patients with mesh plugs and 57 patients with the Prolene Hernia System (PHS) or the Ultrapro Hernia System (UHS) for groin hernia repair. Patients with mesh plug hernia repairs reported higher analgesic usage than those with PHS or UHS (Q08 score: 1.2 vs 1, P=.009), worse impact on health (Q11 score: 1.9 vs 1.5, P=.03), economic burden (Q12 score: 1.4 vs 1.2, P=.04), foreign body sensation (Q15 score: 1.8 vs 1.4, P=.002), discomfort severity (Q17 score: 1.5 vs 1.2, P=.005), less satisfaction in herniorrhaphy (Q18 score: 1.7 vs 1.4, P=.03), less confidence in hernia repair (Q19 score: 2.2 vs 1.9, P=.009), and compromised quality-of-life improvement (Q20 score: 1.4 vs 1.1, P=.03).

## Discussion

### Principal Findings

In this study, we reported the development of a mobile app to facilitate the long-term follow-up of patients with hernia and ascertained the feasibility of the app. The cloud-based system eliminated the need for return visits by subjects who had undergone hernia repairs as early as a decade ago, which, in turn, enhanced the long-term follow-up and outcomes research. Hernia is a type of surgically treated disease with compromised long-term follow-up, as there is neither periodic surveillance nor medication prescription once the defect has been repaired. As well, patients with recurrent disease may seek a second opinion and receive further operations from surgeons in addition to the one resulting in failed repair [13,14]. Therefore, an easy-to-assess reporting system will be of great value for patients to present their immediate abdominal or groin conditions and for surgeons to update treatment outcomes.

For these reasons, we sent invitation letters by post to subjects who had their hernia repaired at our institute more than 1 year ago, with index cases identified from medical records. With an enclosed preset ID and password, invitees could easily download the iOS or Android version of the mobile app, complete the quality-of-life survey, and provide their electrical signature for informed consent within a few minutes. For those not familiar with mobile apps or not equipped with a smartphone, a Google Forms survey provided an online alternative. The response rate was 11.9%, or slightly more than one-tenth of the identified candidates. The majority of the 311 responders were diagnosed with groin hernias, reflecting the clinical scenario of the hernia population.

From our study, the long-term recurrence rate was less than 1%, with 3 patients reporting hernia recurrence. Most of the 311 patients (n=276, 88.7%) reported that they were satisfied or very satisfied with their last hernia repair, 68.5% of patients (n=213) reported no hernia-related discomfort, and 61.1% (n=190) never experienced mesh foreign body sensation. We also compared patients with groin hernias receiving either mesh plug repairs or PHS or UHS repairs and found that those with mesh plugs inevitably experienced more analgesic usage, worse health impaction, economic burden, foreign body sensation, discomfort severity, less satisfaction, less confidence in hernia repair, and compromised quality-of-life improvement. Most importantly, 90.3% of the participants experienced an improvement in quality of life following hernia surgery, indicating that elimination of hernia-related symptoms might be the main contributor to such improvement.

The conceptual structure of the HERQL questionnaire with the postoperative module displayed satisfactory model fit indices (Figure 2), further augmenting the superiority of SEM. Fayers et al [24,25] initiated the efforts to use SEM for the conceptual structure of the instrument used to measure quality of life; they aimed to separate causal variables (ie, symptoms) from effect indicators (ie, functional domains). The critical rationale underpinning the causal-indicative duality was that hernia-associated symptoms impaired subjective perception of quality of life, which was subsequently reflected in functional domain indicator variables as well as in patients’ satisfaction as measured by the postoperative module.

### Comparison With Prior Work

Our study was not the first to perform outcomes research for hernia outside a hospital. Heniford et al [15] conducted outcomes research using the CCS questionnaire, which was mailed to 1048 patients and had a response rate was 12.9%. We invited patients with hernia who had completed hernia repair more than 1 year before the study began, and our response rate was similar to that of the CCS study; however, there was a much longer time interval between herniorrhaphy and questionnaire administration in this study. One major reason for the low response rate was loss of contact due to incorrect addresses, which resulted in undelivered mail. With longer follow-up times, migration could occur naturally and some invitees could pass away; in these cases, patients would become inaccessible. Although compensation was arranged, lack of incentive, worry about fraud, and reluctance to participate might compromise the uptake of the mobile app, constituting another reason for low response. In addition, recall bias did occur, and it was postulated that patients with a recurrence after hernia repair might be more reluctant to participate in this study; consequently, the recurrence rate might be underestimated.

In our previous study, 192 patients who had groin hernias repaired with mesh plugs were compared with 234 patients who had PHS repairs. Postoperatively, the group who had mesh plug repairs had a higher incidence of chronic nondisabling groin pain [33]. In this study, a subgroup analysis was conducted comparing mesh plug repairs with PHS or UHS repairs. Coinciding with our previous study, mesh plugs hampered hernia surgery outcomes with worse symptoms and compromised functionality. It deserves notice that the median follow-up time was only 26.6 months in our previous hospital-based study, which was much shorter than the median follow-up time of 66 months in this study. On the other hand, the HERQL questionnaire validation study was designed with repeated measurements up to 1 year following hernia repair, which is also distinct from the long-term follow-up scope of this study [21]. There are different definitions of chronic pain following hernia repair; we used 1 year as the cutoff for chronic pain, as our previous validation study with repeated measurements indicated that 1 year after hernia repair was a reasonable timepoint for a stable long-term condition.

### Limitations

There were some limitations of this study. First, the retrospective design inevitably introduced recall bias, especially for those with longer follow-up periods. This study evaluated the feasibility of mobile app–based outcomes research, and further study is warranted to eliminate this bias with a more uniform follow-up interval. Second, not all clinical and demographic data were available through chart reviews, such as BMI and fascia defect size, which could hamper post hoc and multivariate analysis considerably. Third, some older adult patients might not be able to complete the survey without an assistant, and there was no printed questionnaire available in the event that the mobile app was not properly installed. Fourth, no further reminder letters were sent by mail and no further phone calls were attempted if there was no response from the initial invitation letter, which inevitably compromised the response rate.

### Conclusions

Knowledge gained from this study can translate into the design of future hernia outcomes research. For example, an updated hernia registry could be established that includes a novel mobile app to enhance the follow-up of patients with hernia, and a cloud-based database could be established for surgeons as well as patients with hernia. A corroborative database would be useful for surgeons to collect clinical and operative details from hernia surgeries, and this would provide a platform for real-time communication between patients with hernia and surgeons and to enhance postoperative follow-up and outcomes assessment. Surgeons could enter the clinical and operative data immediately after completion of hernia repairs using mobile devices, whereas sensitive clinical data would be secured and restricted to authorized personnel. In addition, patients with hernia could review their clinical and operative details in a well-designed and self-explanatory manner. Patients with hernia could also record postoperative events, such as results from a visual analog pain scale, wound condition, and complications, and they could complete the HERQL questionnaire periodically in order to assess the outcomes of hernia repair. Finally, the instant message communication feature can be established to provide an easy and efficient way for patients to report any discomfort to their surgeons, and a proper response from the latter could enhance the long-term follow-up compliance rate of patients with hernia.

The establishment of the mobile app could enhance the quality of care for patients with hernia and facilitate outcomes research for hernia disease with a more comprehensive and complete follow-up, and the feasibility is ascertained herein. The knowledge gained from this project could extended to other common surgical procedures [34,35]. This study will facilitate hernia outcomes research and enhance the quality of care for this common disease by providing a validated HERQL instrument with enhanced sensitivity.

## Appendix

Multimedia Appendix 1 Supplementary materials.

## Abbreviations

AGFI: adjusted goodness-of-fix index
CCS: Carolinas Comfort Scale
GFI: goodness-of-fix index
HERQL: Hernia-Specific Quality-of-Life
PHS: Prolene Hernia System
RMSEA: root mean square error of approximation
SEM: structural equation modeling
UHS: Ultrapro Hernia System
