# Supplementary material for: Long-term Follow-up of Patients With Hernia Using the Hernia-Specific Quality-of-Life Mobile App: Feasibility Questionnaire Study
Source: JMIR Form Res. 2022 Oct 19;6(10):e39759. doi: 10.2196/39759 (PMC9635442; doi:10.2196/39759)
Supplement: Multimedia Appendix 1 [file formative_v6i10e39759_app1.docx]

Multimedia Appendix 1: Supplementary materials


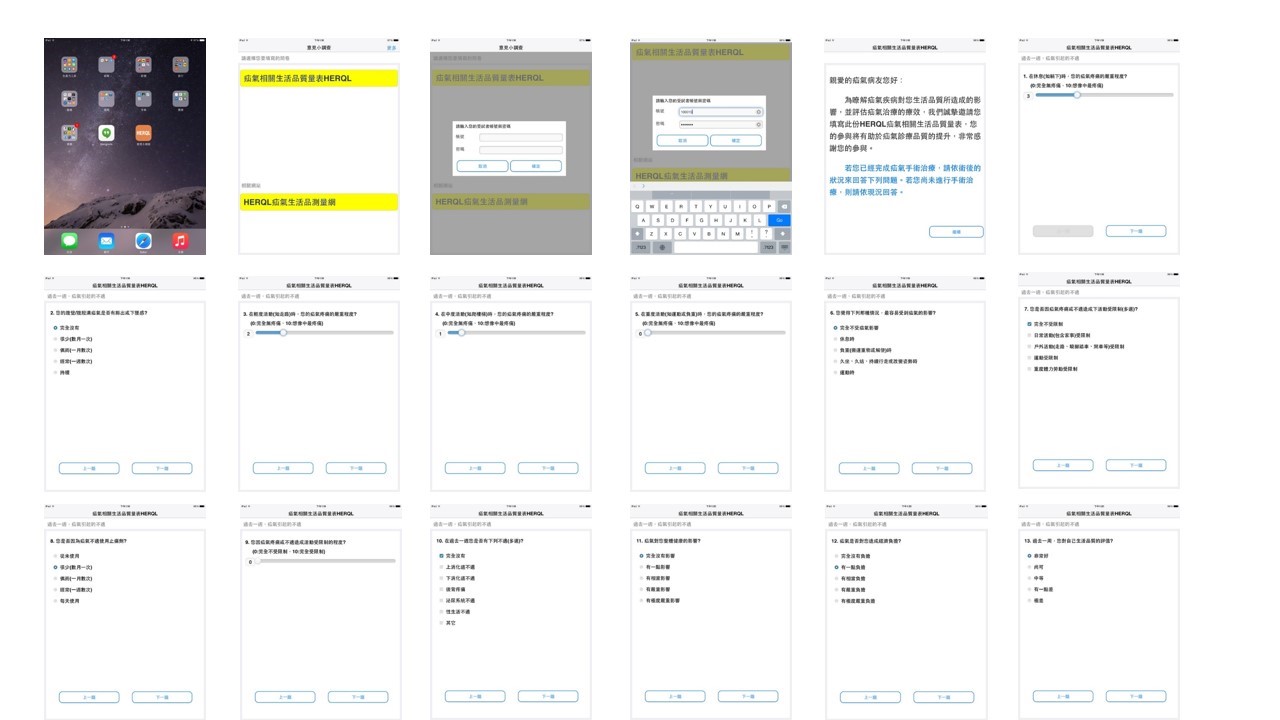


Figure S1. Screenshots of HERQL mobile app from an iOS-based device.


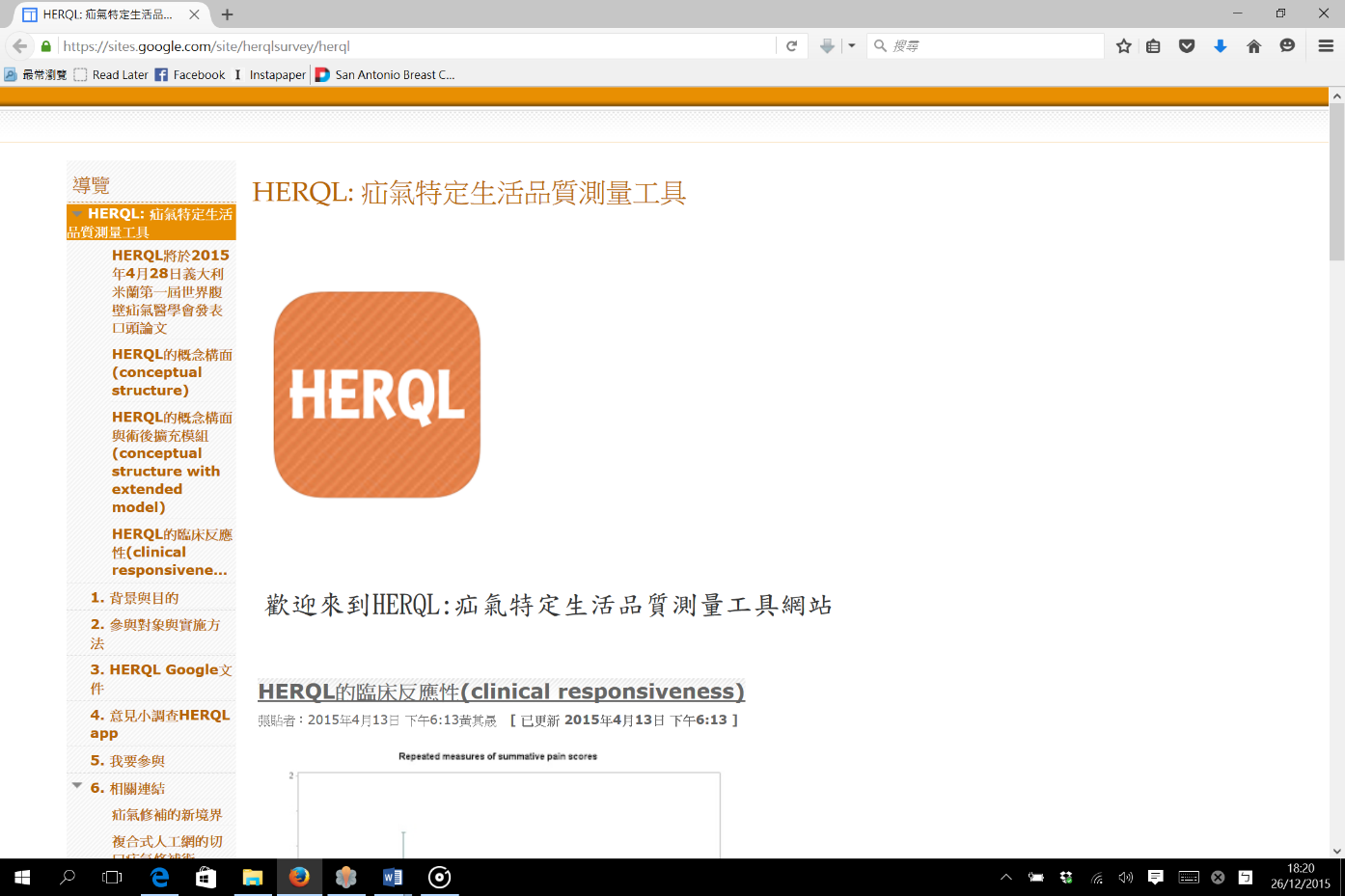


Figure S2. Screenshot for HERQL mobile app google site.


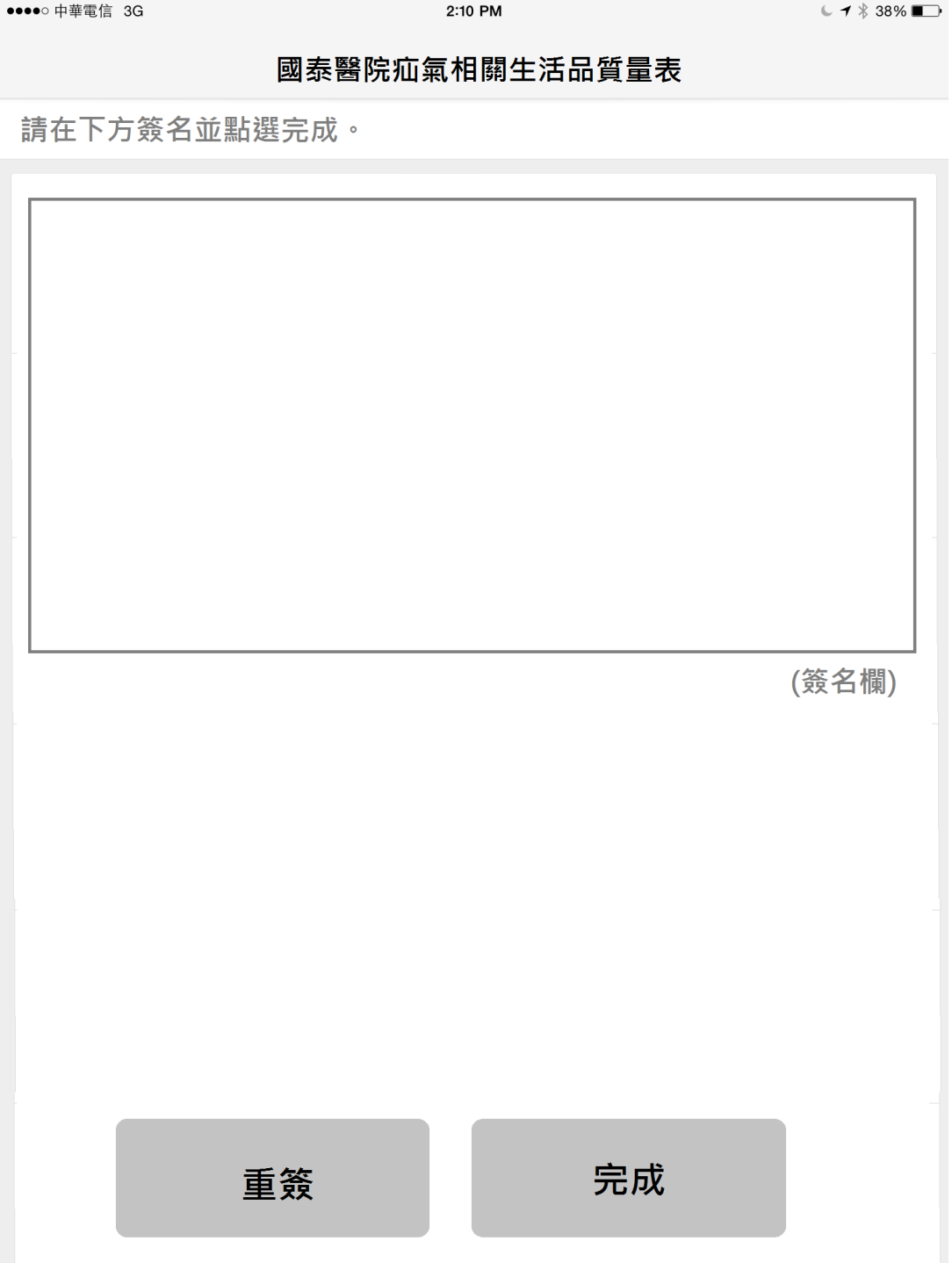


Figure S3. Electrical signature module in portrait mode.
